# Regulation of filial imprinting and structural plasticity by mTORC1 in newborn chickens

**DOI:** 10.1038/s41598-018-26479-1

**Published:** 2018-05-23

**Authors:** Gervasio Batista, Jennifer L. Johnson, Elena Dominguez, Mauro Costa-Mattioli, Jose L. Pena

**Affiliations:** 1 Department of Neuroscience, Albert Einstein College of Medicine, Bronx, New York USA; 2 Department of Neuroscience, Baylor College of Medicine, Houston, Texas USA

## Abstract

Dysregulation of the mechanistic target of rapamycin complex 1 (mTORC1) signaling leads to memory deficits and abnormal social behaviors in adults. However, whether mTORC1 is involved in critical periods of early learning remains largely unexplored. Our study addressed this question by investigating imprinting, a form of learning constrained to a sensitive period that supports filial attachment, in newborn chickens. Imprinting to virtual objects and sounds was assessed after acute manipulations of mTORC1. To further understand the role of mTORC1 during the critical period, structural plasticity was analyzed using DiOlistic labeling of dendritic spines. We found that mTORC1 is required for the emergence of experience-dependent preferences and structural plasticity within brain regions controlling behavior. Furthermore, upon critical period closure, pharmacological activation of the AKT/mTORC1 pathway was sufficient to rescue imprinting across sensory modalities. Thus, our results uncover a novel role of mTORC1 in the formation of imprinted memories and experience-dependent reorganization of neural circuits during a critical period.

## Introduction

Imprinting, the experience-dependent acquisition of early preferences within a sensitive period^1–3^, shapes vital behaviors. In worms, imprinting to different odors underlies aversive and appetitive responses^2,4^. Adult salmons also rely on imprinted odors experienced in early life for navigating towards breeding sites in adulthood^1,5^. In precocial birds, imprinted memories support the rapid development of filial attachment^3,6^. Hence, across evolution, imprinting sculpts reproductive and survival behavioral traits. Understanding the mechanisms involved in this form of time-constrained learning can shed light on the principles controlling behavior and its developmental organization.

The mechanistic target of rapamycin complex 1 (mTORC1) plays a pivotal role in long-term memory formation^7–12^ and different forms of long-term synaptic plasticity^7,13,14^ in the adult brain. Potentiation of hippocampal synapses and memory consolidation in mice requires activation of mTORC1^7^. Similarly, mTORC1-mediated local translation is required for synaptic plasticity in *Aplysia*^15^. The role of mTORC1 in early life is less understood. Yet, recently, it has been reported that mTORC1 signaling supports developmental learning in songbirds^16^. It is thus possible that mTORC1 mediates experience-dependent reorganization of neural circuits within critical periods. Here we asked if mTORC1-mediated plasticity is relevant for imprinting. To address this question, we investigated the role of mTORC1 in the formation of imprinted memories of newborn chickens where mTORC1-mediated plasticity can be tracked to specific brain regions controlling behavior.

In chickens, protein synthesis is required for the consolidation of imprinted memories^17,18^. Auditory imprinting, the acquisition of a preference for a given sound during the critical period^19^, is controlled by dephosphorylation of the eukaryotic translation initiation factor subunit 2 alpha (eIF2α)^18^. In contrast, the mechanisms regulating experience-dependent regulation of protein synthesis in visual imprinting remain unknown. The mTORC1 complex appears a good candidate to carry out this function given that it regulates translation through three of its downstream targets: the ribosomal protein S6 kinase (S6K)^20^, the eukaryotic translation initiation factor 4E-binding proteins (4E-BPs)^21^ and the eukaryotic elongation factor 2 kinase (eEF2K)^22,23^. Because of its relevance in memory formation in adulthood^22,24^ and its role in experience-dependent translation^22,25^, mTORC1 activation might underlie imprinting across sensory modalities.

We found that mTORC1 is recruited within the mediorostral nidopallium/mesopallium (MNM)^26,27^ and the intermediate medial mesopallium (IMM)^28–30^, forebrain regions involved in auditory and visual imprinting, respectively. This endogenous activation of mTORC1 was required for the acquisition of imprinted preferences and dendritic spine plasticity. On the other hand, acute pharmacological enhancement of mTORC1 signaling was able to recover imprinting beyond the critical period. These results provide evidence for a previously unknown role of mTORC1 in filial imprinting and reveal that targeting mTORC1 may rejuvenate plasticity upon critical period closure.

## Results

mTORC1 controls long-term synaptic plasticity and memory consolidation^24^. Yet a major outstanding question is whether mTORC1 regulates critical periods in early life. Involvement of mTORC1 in early learning has been shown in juvenile birds, where mTORC1 is required for song learning^16^. Here, taking advantage of the precocial behavior of newborn chickens and rapid onset of imprinting, we addressed the role of mTORC1 during the critical period for imprinting by combining biochemical assays to assess endogenous activation of mTORC1, behavioral pharmacology and analysis of structural changes correlated to the learning process. In addition, we recovered the imprinting upon closure of the critical period.

### Experience-dependent activation of mTORC1

**Figure 1:**
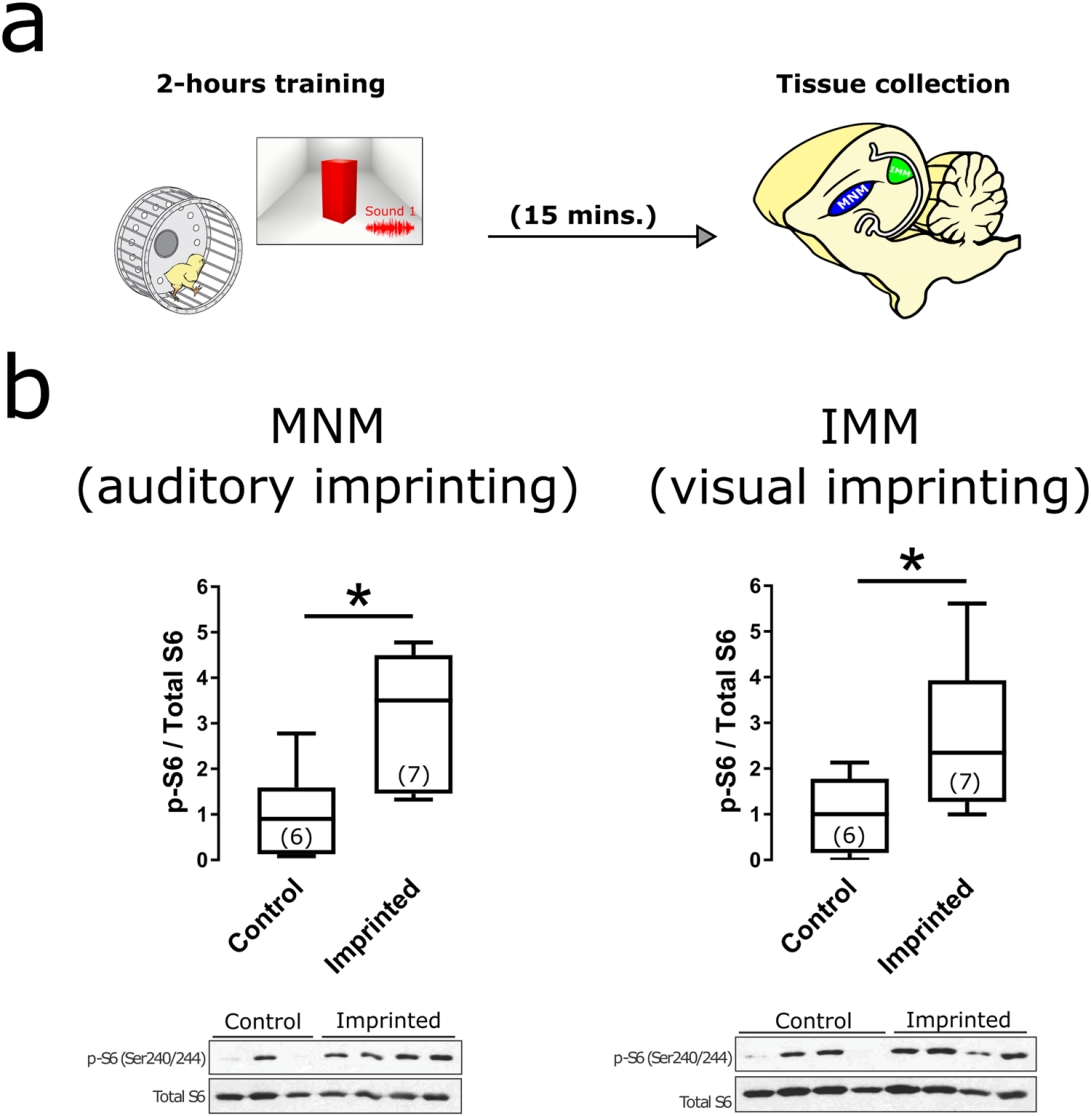
Experience-dependent activation of mTORC1. (**a**) Tissue samples from IMM (green) and MNM (blue) were collected for western blotting, 15 minutes after training. (**b**) Phosphorylation of the S6 ribosomal protein in MNM (left) and IMM (right) was increased in trained (N = 7) chickens compared to controls (N = 6). Representative western blots are shown below each panel. Box plots show mean and interquartile. Error bars show 10–90 percentile, *indicates p < 0.05 from two-sample t-test. The number of animals used in each group is listed inside parenthesis. (Illustrations by Michael Beckert).

We first verified if mTORC1 was activated by training in MNM and IMM, forebrain areas crucial for auditory and visual imprinting, respectively^3,26,30^. While there are different read-outs of mTORC1 activation (e.g. 4E-BP phosphorylation), phosphorylation of the ribosomal protein S6 (p-S6) was chosen because it is highly conserved and has been previously used to assess learning-dependent activation of mTORC1 in mice^7^ and birds^16^. Endogenous activation of mTORC1 after training was investigated in both MNM and IMM (Fig. 1a), forebrain structures involved in auditory and visual imprinting, respectively^31,32^.

Western blots from MNM samples showed increased activation of mTORC1 compared to untrained animals, as indicated by the increased p-s6/total-S6 ratio (Fig. 1b) (Two-sample t-test, p = 0.0176). Thus, training activated mTORC1 signaling in the auditory imprinting-relevant area MNM. This effect could not be explained only by lack of movement in control animals since only chickens that significantly moved the wheel within the first half hour of training were included in the sample.

In addition, we examined mTORC1 activation in IMM, an area involved in visual imprinting^3^. Consistently, IMM samples from trained chickens also showed higher p-S6/total-S6 ratios than control animals (Fig. 1b) (Two-sample t-test, p = 0.0348). Therefore, the training method used to induce imprinting led to mTORC1 activation in both auditory and visual imprinting areas.

### mTORC1 signaling is required for imprinting

**Figure 2:**
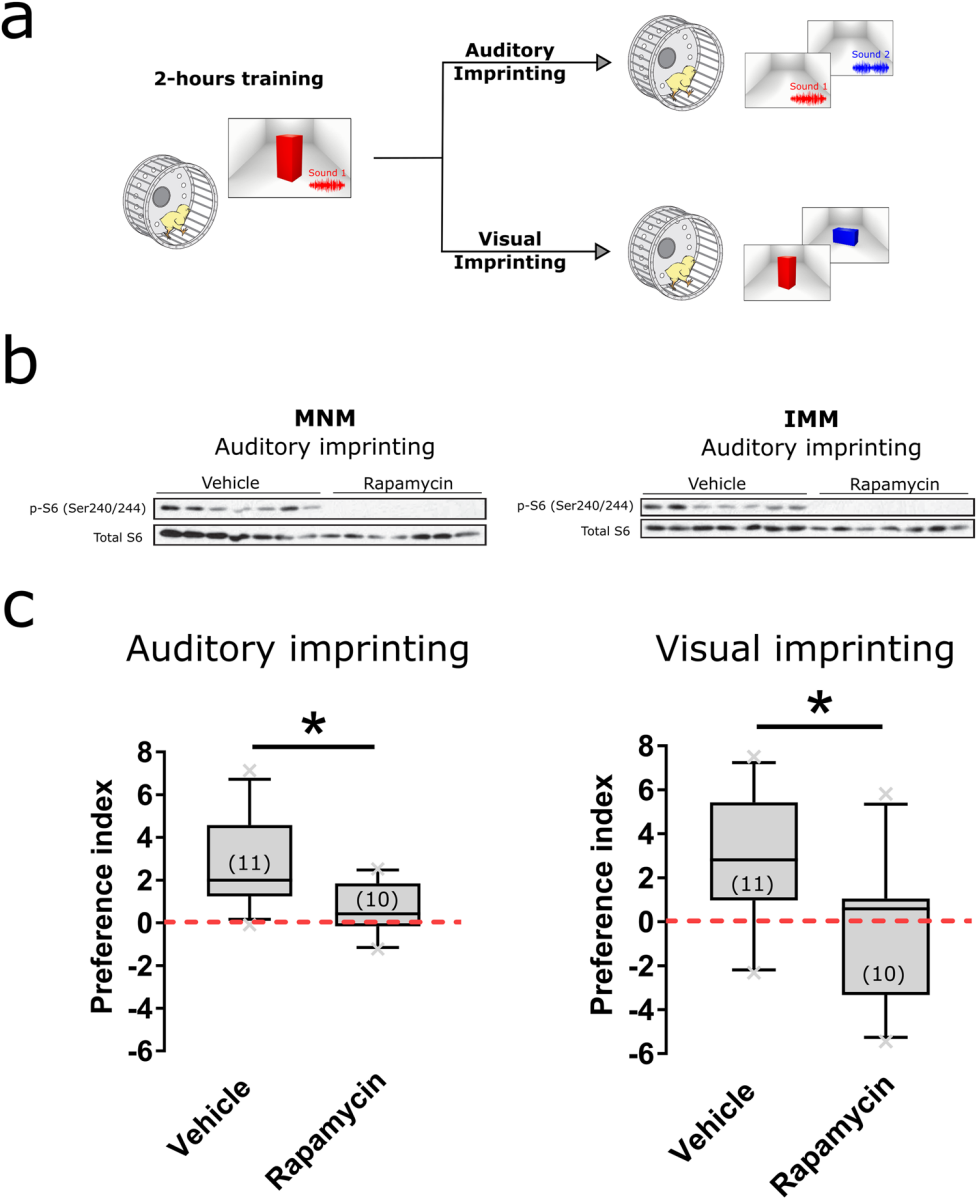
mTORC1 blockade impairs auditory and visual imprinting. (**a**) Chickens were trained for two hours and injected either with rapamycin (N = 11) or vehicle (N = 10) prior to training. Visual and auditory imprinting were separately tested the day after training. (**b**) Rapamycin (N = 7) strongly reduced phosphorylation of S6 within MNM (left) and IMM (right) compared to vehicle-injected chickens (N = 7). (**c**) Vehicle- (N = 11), but not rapamycin-injected (N = 10) chickens showed auditory (left) and visual (right) imprinting the day after training. Box plots show mean and interquartile. Error bars show 10–90 percentile, *indicates p < 0.05 from two-sample t-test. Number of animals used for each group is inside parenthesis.(Illustrations by Michael Beckert).

We tested whether inactivation of mTORC1 affects memory formation specifically. Imprinting develops rapidly (2-hour training), which allows acute pharmacological manipulations, reducing the potential confound of compensatory mechanisms induced by prolonged treatments. Taking advantage of these features we investigated whether activation of mTORC1 supports behavioral and structural plasticity in imprinting. To investigate whether mTORC1 activation is required for the formation of imprinted memories, we injected the mTORC1 inhibitor rapamycin to chickens, prior to training (Fig. 2a,b).

Consistently with our western blot analysis, mTORC1 blockade abolished both auditory and visual imprinting (Fig. 2c) (Two sample t-test, p_Auditory-imprinting_ = 0.0067, p_Visual-imprinting_ = 0.0363). Chicks treated with rapamycin did not show a preference for the sound experienced during training (Fig. 2c). In contrast, vehicle-treated animals showed auditory imprinting 24 hours after training (Fig. 2c). Rapamycin also blocked visual imprinting (Fig. 2c). On the other hand, chickens injected with vehicle developed a normal preference for the object presented during training. Importantly, no change in basal locomotion was observed between vehicle- and rapamycin-injected chickens (Supplementary Fig. S1a). Together our results demonstrate that long-term auditory and visual imprinting requires mTORC1 activation during the critical period.

### mTORC1-mediated structural plasticity

**Figure 3:**
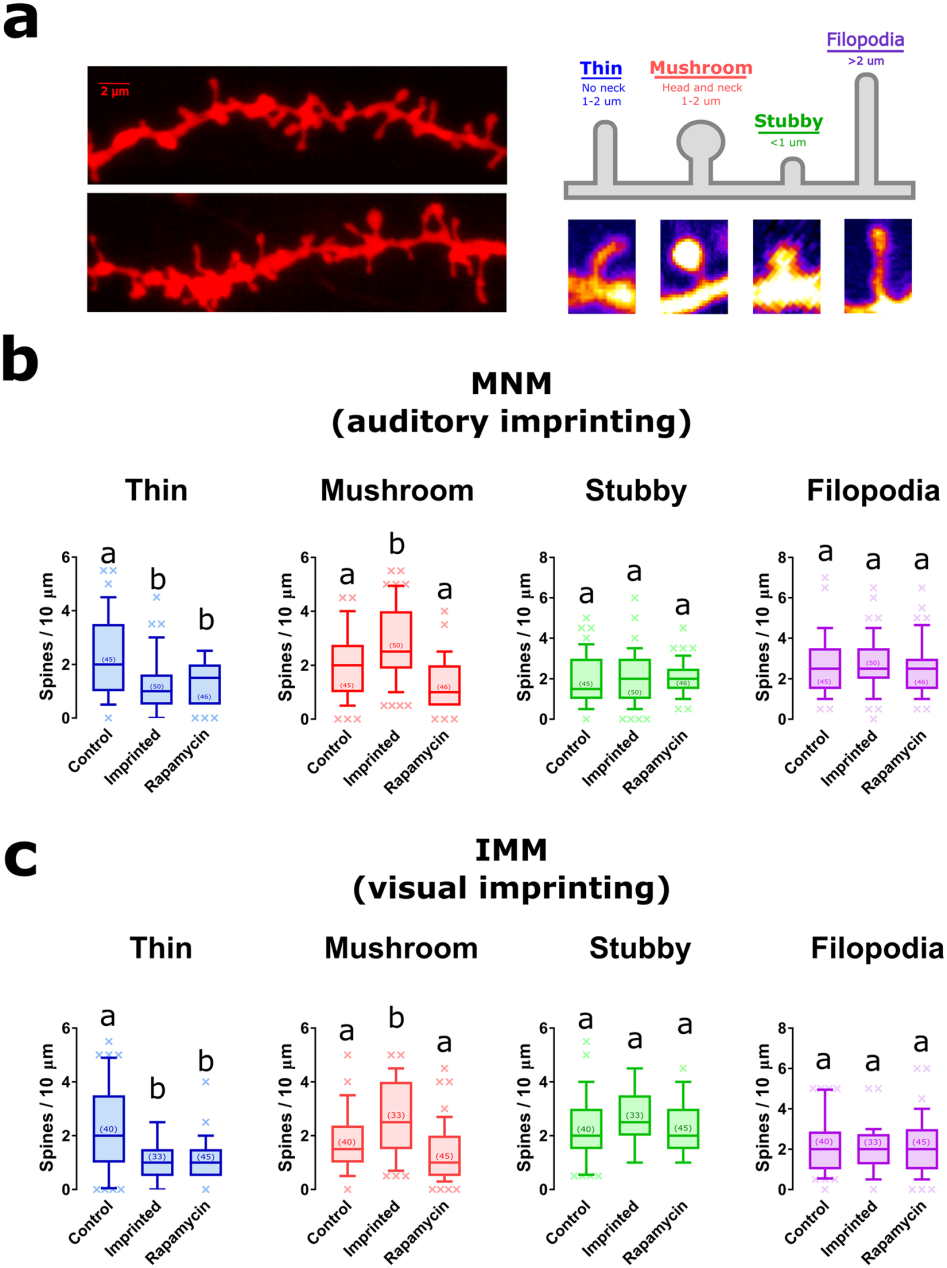
Structural plasticity in MNM and IMM is disrupted by mTORC1 inactivation. (**a**) Spines were imaged using DiOlistic labeling and confocal imaging (left) to image dendrites in IMM (top, left) and MNM (bottom, left). Dendritic spines were classified in thin, mushroom, stubby and filopodia based on length and presence or absence of a neck (top, right). Different groups can be distinguished under confocal imaging (bottom, right). (**b**,**c**) Imprinted chickens show a decrease in thin spines (blue) and an increase in mushroom spines (red) compared to control animals. Structural plasticity of mushroom spines was blocked in chickens treated with rapamycin. No changes in stubby (purple) or filopodia (green) were detected. Box plots show mean and interquartile. Error bars show 10–90 percentile; different letters indicate statistically significant differences (p < 0.05) between groups from Kruskal-Wallis test, Dunn’s multiple comparisons test. Number of dendrites analyzed for each group is inside parenthesis.

Long-term changes in the organization of neural circuits are hypothesized to support memory storage^33^. Hence, to shed light on how mTORC1 could underlie imprinting, we analyzed experience-dependent alterations in spine morphology, the cellular compartment receiving most excitatory inputs^34^. To achieve this, we combined DiOlistic labeling and confocal imaging of dendrites within MNM and IMM (Fig. 3a).

Because spine morphology is informative about the functional state of the embedded synapses^34^, we classified them in stubby, filopodia, thin and mushroom spine types (Fig. 3). To test whether structural plasticity of spines required mTORC1 signaling, we injected chickens with either vehicle or rapamycin prior to training and tissue processing.

As previously shown^18^, imprinting triggered an increase in the number of mushroom spines in MNM and IMM that was paralleled with a reduction in thin spines. In MNM, rapamycin blocked the increase in mushroom spines (Fig. 3b) (Kruskal-Wallis test, Dunn’s multiple comparisons test, p < 0.001). However, the reduction in the number of thin spines after training was not blocked by rapamycin (Fig. 3b) (Kruskal-Wallis test, Dunn’s multiple comparisons test, p = 0.037). On the other hand, no changes in filopodia or stubby spines were detected in rapamycin-treated animals or controls.

Rapamycin injection also blocked structural plasticity in IMM. In chickens where mTORC1 was inactivated, IMM neurons did not exhibit an increase in the number of mushroom spines (Fig. 3c) (Kruskal-Wallis test, Dunn’s multiple comparisons test, p < 0.001) compared to the control group. Yet, as in MNM, rapamycin-treated chickens showed fewer thin spines after training, compared to untrained ones (Fig. 3c) (Kruskal-Wallis test, Dunn’s multiple comparisons test, p < 0.01). No significant changes in the number of stubby or filopodia spines were observed after training in IMM (Fig. 3b,c). These results indicate that mTORC1 activation is required for structural plasticity in both MNM and IMM.

### mTORC1 activation is recovers imprinting after closure of the critical period

**Figure 4:**
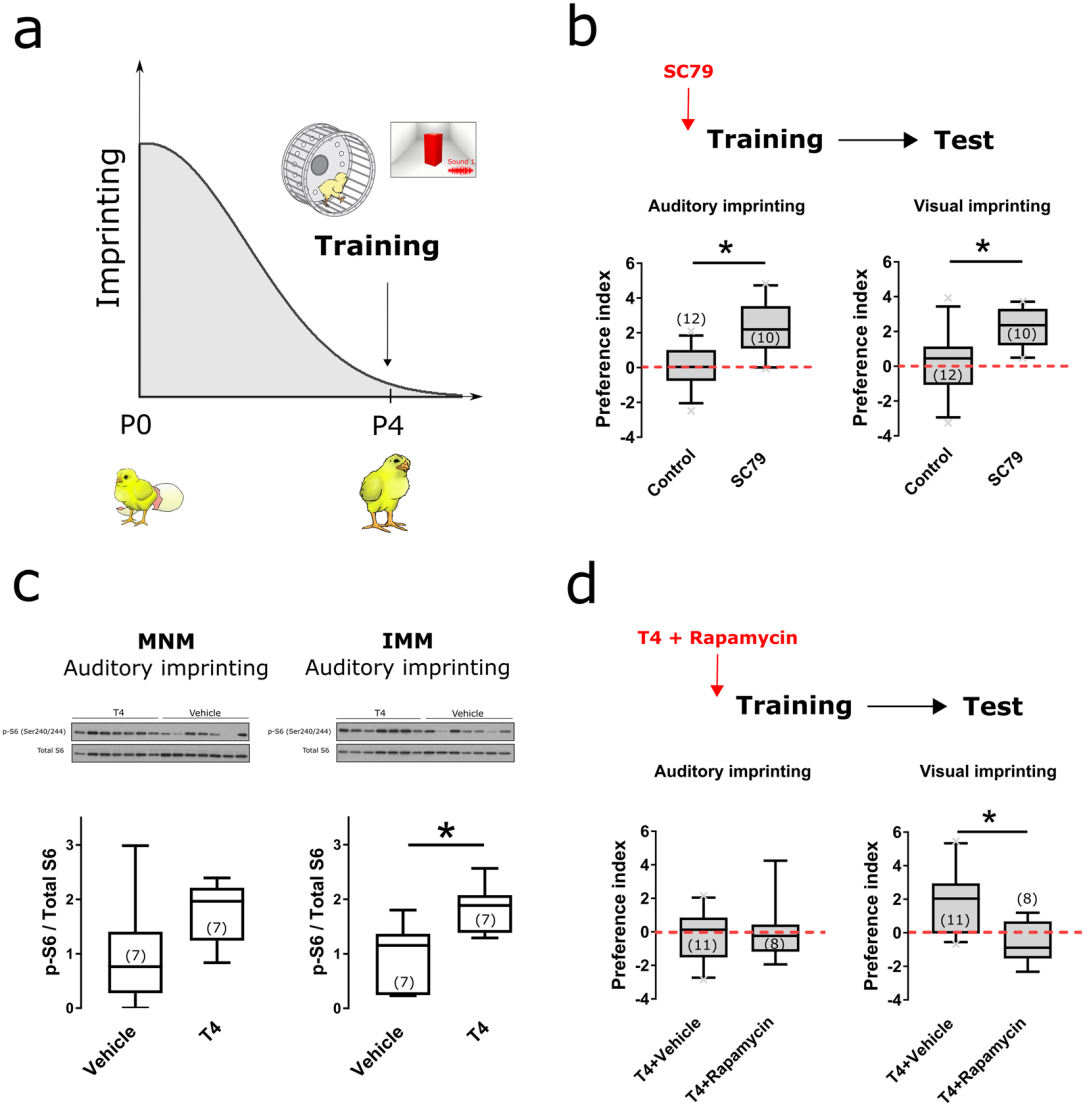
mTORC1 underlies recovering of imprinting upon closure of the critical period. (**a**) Chickens were trained on P4, when the critical period is closed and mTORC1 activation was manipulated to assess its role in the critical period. (**b**) Chickens were injected with the AKT activator SC79 before training. Activation of mTORC1 restored both auditory (left) and visual (right) imprinting (N_Control_ = 12, N_SC79_ = 10). (**c**) Injections of the thyroid hormone thyroxine (T4) enhanced mTORC1 signaling in IMM (right) but not MNM (left) (N_Vehicle_ = 7, N_T4_ = 7). (**d**) Injections of T4 on P4 had no effect on auditory imprinting (left). Re-opening the critical period for visual imprinting by thyroid hormone (T4) also requires mTORC1 activation (right) (N_T4_ = 11, N_T4+Rapamycin_ = 8). Box plots show mean and interquartile. Error bars show 10–90 percentile, *indicates p < 0.05 from two-sample t-test. Number of animals used for each group is inside parenthesis (Illustrations by Michael Beckert).

Because blockade of mTORC1 impaired both behavioral and structural plasticity, we aimed to test whether enhancing mTORC1 activity could restore plasticity upon closure of the critical period (Fig. 4a). Four-day old chickens, which are unable to become imprinted^18,29^, were injected with the AKT activator SC79^16,35^. We found that this manipulation recovered imprinting in both sensory modalities (Fig. 4b) (Two sample t-test, p_Auditory-imprinting_ = 0.0015, p_Visual-imprinting_ = 0.0085). These results indicate that mTORC1 activation is sufficient to restore plasticity upon critical period closure. No significant difference in basal locomotion between vehicle and SC79-injected groups was detected (Supplementary Fig. S1b).

To further examine the ability to recover imprinting after the critical period by manipulation of the mTORC1 pathway, we explored other potential ways for mTORC1 activation. Thyroid hormones, in particular thyroxine (T4), have been shown to restore visual imprinting in P4^29^. As shown in Fig. 4c, systemic injections of T4 strongly activated mTORC1 in IMM but not significantly in MNM (Two sample t-test, p_IMM_ = 0.0092, p_MNM_ = 0.10). Thus, we asked whether mTORC1 was required for the T4-dependent reopening of the critical period for visual imprinting. In fact, T4 rescued visual imprinting in P4 chickens but did not affect auditory imprinting (Fig. 4d). Remarkably, T4-dependent recovery of visual imprinting was abolished if mTORC1 was inactivated with rapamycin (Fig. 4d) (Two sample t-test, p = 0.0037). Basal locomotion was not different across experimental groups (Supplementary Fig. S1c).

Together these findings demonstrate the requirement of mTORC1 in auditory and visual imprinting. Furthermore, we show that pharmacological enhancement of mTORC1 could be a novel strategy to reopen critical periods.

## Discussion

The mechanistic target of rapamycin complex 1 (mTORC1) regulates a variety of cellular functions, ranging from autophagy to proliferation^21^. Activation of this protein complex in the brain mediates neuronal plasticity^7,14,36,37^, long-term memory formation^7,8,38^, biological rhythms^39,40^ and social behaviors^41–43^. While most studies aimed to uncover mTORC1 function in the adult brain, here we demonstrated that mTORC1 activation is required for imprinting and circuit re-organization during a critical period in newborn chicks. Furthermore, we showed that imprinting can be recovered, once the critical period is closed, through acute enhancement of mTORC1 signaling.

### mTORC1 is recruited within imprinted circuits

Western blot analysis showed that exposure to virtual objects and artificial sounds activated mTORC1 in forebrain areas relevant for visual and auditory imprinting. This activation was required for visual and auditory, imprinting. However, our approach is not able to identify the contribution of specific neuronal types to the observed increase in the p-S6/Total-S6 ratio, which could contribute to variability across samples. Future studies should target mTORC1 activation within specific cell-types (e.g. inhibitory interneurons). This might be particularly important given that, as shown in the visual cortex of mice^44^, maturation of parvalbumin positive (PV) cells sets the onset of sensitive periods. Similarly, these neurons seem to be also important for imprinting. An experience-dependent increase in the number of PV-cells after filial imprinting has been reported^45^. It is possible that mTORC1 is heavily involved in the maturation of PV-cells. This hypothesis is likely since genetic ablation of PTEN, an inhibitor of mTORC1 activation, increases the proportion of PV-neurons in the mouse neocortex^46^.

mTORC1 mediates structural plasticity of dendritic spines, the main site receiving excitatory inputs in neurons^34^. Changes in spine morphology are tightly associated with potentiation and depression of synaptic function^47^ and learning experiences^48^. These changes are indicative of circuit reorganization^48^. In particular, spine enlargement and acquisition of mushroom shape, is associated with increased AMPA currents^49^ and longer spine stability^50,51^. We found that rapamycin injections blocked the increase in mushroom spines detected 24 hours after imprinting training in both MNM and IMM. Similarly, mTORC1 appears to mediate spine plasticity in cultured neurons^52^. While spine plasticity might suggest that mTORC1-mediated post-synaptic changes are important for imprinting, mTORC1 could also be a key player in regulating pre-synaptic processes, as shown previously in the hippocampus of mice^14^. Notably, training triggered a reduction in the number of thin spines. This process resonates with a form of homeostatic plasticity described in the hippocampus^53^, where potentiation of a subset of spines is counterbalanced by synapse elimination within the same dendrites. In our case, the experience-dependent reduction of thin spines was not blocked by mTORC1 inactivation, suggesting that this process is mTORC1 independent. It would be interesting to assess if in the hippocampus, rapamycin blocks spine potentiation but not synapse elimination. This might be the case since manipulation of mTORC1 in cultured neurons particularly affects structural plasticity of mushroom spines but not of other subtypes^52^.

### Protein synthesis regulation by mTROC1: a potential downstream mechanism underlying imprinting

The downstream mechanisms controlled by mTORC1 to support imprinting remain unclear. However, it is likely that mTORC1-mediated protein synthesis, through S6K, 4E-BPs and eEF2K^21,24^, underlies critical period plasticity. mTORC1 phosphorylates S6K and 4E-BPs to regulate translation initiation^21,54^, a mechanism known to mediate memory consolidation in adults^7,22^. On the other hand, mTORC1 can control the elongation of nascent polypeptide chains through phosphorylation of eEFK2^15^. In *Aplysia*, the elongation factor 2 (eEF2) supports long-term facilitation of synaptic function even when translation initiation is blocked^25^. Because of its potential for controlling translation at different levels, mTORC1 might be a ‘master’ regulator of experience-dependent protein synthesis necessary for imprinting. In contrast, other translational control mechanisms involved in imprinting, such as dephosphorylation of the translation initiation factor eIF2α^18^, may have more subtle and specific roles. For instance, eIF2α-mediated translation regulates auditory, but not visual, imprinting^18^. To better understand how experience recruits eIF2α and mTORC1 within imprinted circuits, species tractable by genetic approaches might be required, such as C. elegans, where imprinting has also been shown^2,4^.

### Re-opening critical periods through translational control mechanisms

Mechanisms controlling experience-dependent protein synthesis during critical periods can offer novel routes to rejuvenate plasticity. Enhancing eIF2α-mediated translation, for example, can recover auditory imprinting in chickens after the critical period^18^. Since mTORC1 can control protein synthesis, its manipulation could hold promise for restoring critical period plasticity across species. Consolidation of ocular dominant plasticity (ODP), the experience-dependent reorganization of visual binocular responses in the primary visual cortex (V1) of mammals, requires mTORC1 activation during sleep^55^. However, no attempt to recover ODP through mTORC1 activation after critical period closure has been pursued^55^. Our results demonstrate that direct enhancement of AKT/mTORC1 signaling is sufficient to restore visual and auditory imprinting upon critical period closure. Yet, these data cannot rule out that AKT activation by SC79 restores imprinting through downstream targets other than mTORC1. In addition, we showed that mTORC1 is recruited in the reopening of the critical period by thyroxine, one of the thyroid hormones capable of restoring visual imprinting^29^. Interestingly, during the critical period for birdsong acquisition in zebra finches, thyroid hormones levels increase^56^, as well as mTORC1 activation in response to the tutor’s song^16^. Our results raise the intriguing question of whether thyroid hormones also engage mTORC1 in songbirds to support behavioral plasticity. It remains untested whether activation of mTORC1 can rescue song learning outside of the critical period.

### mTORC1 as key regulator of complex behaviors during development

In addition to its role in imprinting, mTORC1 may be important in time-constrained learning supporting the development of other brain functions, such as social behaviors. Yet the lack of early behavioral readouts in most vertebrates has stalled progress in understanding the early roles of mTORC1. Recently, in one of the few studies investigating mTORC1 in juveniles, it was shown that repeated inactivation of mTORC1 in songbirds impairs acquisition of the tutor’s song^16^. This result is consistent with our findings showing that rapamycin injections block imprinting. However, while acute rapamycin administration inactivates mTORC1^57^, repeated exposure to rapamycin also reduces the activity of the mTOR complex 2 (mTORC2)^58^, involved in actin remodeling regulation^59,60^. Thus, in the case of songbird learning^16^ it has no yet been ruled out that both mTOR complexes have a role in the behavioral plasticity during the critical period. The single-trial nature of imprinting in chickens allowed us to acutely perform gain- and loss-of-function manipulations on mTORC1, which is less likely to affect mTORC2 signaling. Yet, mTORC2 might still play a role in imprinting. To address this important question, specific genetic manipulations and pharmacological tools will be required.

## Conclusions

Together, our results demonstrate that mTORC1 mediates behavioral and structural plasticity in newborn chickens, supporting the idea that mTORC1 controls the storage of long-term memories since birth. We also showed that pharmacological activation of mTORC1 could help to reinstruct brain function after critical windows are closed, opening a potential avenue for therapeutic interventions to recover brain plasticity.

## Methods

### Animals

White Leghorn chicken eggs (E14-17) were received from a vendor (Charles River supplier) every week and incubated (Grumbach incubator, compact S84) at 37–38 °C in darkness until hatching. Humidity and temperature were constantly monitored to ensure proper embryonic development and normal hatching. On hatching day, chicks were transferred to individual compartments inside a brooder (Brinsea TLC-5). Temperature was kept at 37–38 C, and chickens were reared in the dark until the time of the experiments. Because chickens are capable of eating and drinking in darkness, water and food was provided *ad libitum*. This method of rearing does not affect health, locomotion and sensory acuity of chicks^29^. All the experiments were approved by the institutional animal care committee (IACUC) at Albert Einstein College of Medicine (protocol 20140910). All experiments were performed in accordance with relevant guidelines and regulations.

### Imprinting training and preference test

Experiments and analysis described in this section were performed blind to treatment, between 8 AM to 6 PM. Both female and male chicks were used. In this study these data were pooled. To isolate chickens from environmental noise, training and tests were carried inside a sound proof chamber (IAC acoustics) at 37 °C. The day of the experiment, subjects were first placed under a white light for 30 minutes. This step has been proven to enhance imprinting training efficiency^61^. After priming with light, chickens were moved inside a running wheel equipped with a ring of magnets to assess locomotion (Med Associates). The wheel (internal diameter = 18 cm) was connected to a counter and a motherboard (Med Associates, DIG-700G, DIG-726). Data was stored in a computer for offline analysis.

In front of the running wheel, a monitor (ACER LCD, 17′′) and a speaker were placed. Videos of animated figures, blue or red, were generated using the open source program Blender (http://www.blender.org/). Animated objects were designed to move inside a virtual environment. Both animated objects covered the same area and followed identical trajectories on the screen. Expansion and contraction animation effects were added, matching the timing of sounds employed to test auditory imprinting. Two distinct sounds (labeled ‘sound 1’ and ‘sound 2’) were generated using Audacity software (2.1.0). The frequency range (0–3 KHz) was the same for all sounds, within the chickens’ audible range. Sound 1 contained sharp frequency steps and sound 2 frequency sweeps (ramps). A command script written in Matlab controlled the Med Associates equipment through a USB DAQ card (National instruments USB-6008). Each sound was played 12 times per minute, with an inter-stimulus interval of 3 seconds.

Training consisted in the presentation of audiovisual stimuli in bouts of 4 minutes with an inter-bout interval of 1 minute. During intervals, the speaker remained silent and the room was in complete darkness. If chickens were inactive within the first half-hour of the training, they were not considered for this study (N = 3). Training length was 120 minutes for all experiments and imprinting was tested one day after training.

Independent assessment of auditory and visual imprinting was achieved with a sequential test, where novel and imprinted stimuli were presented in alternation, in contrast to other studies that have used a simultaneous choice test^29^. The presentation of stimuli in sequence during tests allowed us to randomize stimulus presentation, measure baseline locomotion and evaluate the response to novel and imprinted stimuli independently. Each test included 5 presentations of the imprinted stimulus and 5 presentations of the novel stimulus. The duration of each presentation was 1 minute. The amount of locomotion inside the running wheel when no stimulus was presented, was measured during 30-second time windows immediately preceding each trial. This value, referred here to as baseline locomotion, was included in the preference score metric as a normalization factor. Imprinted and novel stimuli were alternated over 5 consecutive blocks. The first stimulus that started the sequence was picked randomly. Randomization of stimulus presentations prevents the emergence of biases and motivation changes over time. Importantly, no innate biases for the stimuli used was detected (Supplementary Fig. S2).

Our computation of the preference indexes normalized differences between locomotion to novel and imprinted stimuli by the average baseline locomotion in the wheel when no stimulus was presented. Therefore, to measure imprinting strength, we calculated a preference index (PI), *PI* = ∑*(Imprinted*_*STL*_ − *Novel*_*STL*_)/*Baseline*_*A*_ where *STL* indicates stimulus-triggered locomotion either during presentation of the imprinted stimulus (*Imprinted*_*STL*_) or presentation of the novel stimulus (*Novel*_*STL*_), and baseline_A_ refers to the average baseline locomotion across the experiment. An advantage of this quantification over previous methods is that: (1) it considers basal locomotion before each stimulus presentation, and (2) it normalizes locomotion during stimulus presentation trials by average locomotion.

### Pharmacological manipulation of mTORC1 activation

Rapamycin specifically blocks mTORC1^7,8^. We used a rapamycin dosage shown to impair LTM when injected systemically before a 2-hour training (20 mg/Kg^62^, N = 10) diluted in 4% ethanol, 5% Tween-80 and 5% PEG-400. We confirmed mTORC1 blockade by rapamycin with Western blotting (Fig. 2b). Behavioral measures of imprinting were performed 24 hours after training. Vehicle-injected chicks were used for comparison (N = 11).

To enhance mTORC1 signaling outside of the critical period (P4) we employed two pharmacological agents: (1) we used the AKT activatorSC79^16,35,63^ (0.04 mg/g, N = 10) diluted in diluted in 5% Tween-80 and 5% PEG-400 to enhance mTORC1 activity; and (2) Thyroxine (30 µg/100 gr, N = 11), which is known to extend the sensitive period for visual imprinting^29^, after verification that such manipulation strongly activates mTORC1 within IMM (Fig. 4c). SC79-treated group was compared with vehicle-injected chickens (N = 12). To evaluate if the previously reported^29^ T4-mediated re-opening of the critical period requires mTORC1 activation, we administered both T4 and rapamycin (N = 8) to P4 chicks before training. All drugs used in our study were injected systemically (i.p.) 30 minutes prior to training.

### Western blotting

Lysates of IMM and MNM (anatomical boundaries described below) were obtained from brain tissue, punched out from 0.75- to 1 mm-thick sagittal brain slices collected from imprinted (N = 7) and control (N = 6) animals. We used antibodies against the phosphorylated ribosomal protein p-s6 (Cell Signaling Cat #2215) and total s6 protein (Cell Signaling Cat #2217), following standard protocols described before^64^. Control tissue samples were obtained from chickens that ran on the wheel towards a screen displaying only a static image of an empty room.

### Dendritic spine analysis

Chicks were euthanized, and brains were rapidly removed and sank in PFA (4%) for 1 hour before being transferred to PBS. Fixation period was set to 1 hour because over-fixation damages the cellular membrane, affecting DiI spread. Sagittal slices (200 µM thick) were made with a vibratome (Leica VT 1000S). The DiOlistic technique was used to obtain sparse labeling of neurons. In brief, tungsten beads coated with a lipophilic dye (DiI) were delivered with a modified gene gun. After allowing the DiI to spread for 24 hours at room temperature, slices were mounted using ProLong Gold mounting media. Confocal imaging (Zeiss LSM 510 Meta Duo V2) was used to collect Z-stacks (63×, zoom 3) from dendritic arbors of MNM and IMM neurons. The criteria to define regions of interest within a dendrite were based on distance from the soma (50–75 µm), always after the first dendritic bifurcation.

All images were taken blind to experimental groups and treatment. IMM is located 2.5 mm from the dorsal surface of the forebrain and 0.5–1 mm from the caudal edge, limited below and laterally by the lateral ventricle. MNM stereotaxic coordinates are: 0.5–1 mm lateral from the midline, 3 mm ventral from the dorsal surface of the brain and 5 mm rostral from the caudal edge of the forebrain, below the lateral pallial lamina that separates the hyperpallium and mesopallium^65^. All compared samples were processed the same day, using the same protocol, and images were taken with equal microscope settings. As in other comparative testing, control animals were housed in the same conditions as trained animals but presented with a screen displaying the static image of a virtual empty room.

Image J software (Version 1.50a) was used to count spines in each group by a subject blind to treatments. To enhance spine contrast, a multicolored lookup table (Fire) was used. Two 10 μm segments were marked randomly along each secondary dendritic branch. Spines along each of the two segments were counted by a blind experimenter. The spines‘ head width, presence of neck and overall length were used for classifying them in stubby (length <1 μm), thin (1 <length <2 μm, no clear head or neck), mushroom (1< length <2 μm, head and neck) and filopodia (length >2 μm)^34,66,67^.

### Statistical analyses

SigmaPlot (Systat Software) was used for statistical analysis. Data distribution normality was assessed using the Shapiro-Wilk and F-test to evaluate differences in variance. When variance was significantly different across datasets, the Welch’s correction was used. Statistics were based on the two-sided Student’s t test, or the two-way ANOVA and Bonferroni post-hoc test for multiple comparisons of normally distributed samples. Otherwise the Mann-Whitney or the Kruskal-Wallis and Dunn’s multiple comparisons tests were used. Within-group variation is indicated by box plots showing the mean, interquartile and 10–90 percentiles (error bars) for each distribution. p < 0.05 was the criterion for significance.

## Electronic supplementary material

Supplementary figure
