# Supplementary material for: Regulation of filial imprinting and structural plasticity by mTORC1 in newborn chickens
Source: Sci Rep. 2018 May 23;8:8044. doi: 10.1038/s41598-018-26479-1 (PMC5966437; doi:10.1038/s41598-018-26479-1)
Supplement: Supplementary file 1 — Supplementary figure [file 41598_2018_26479_MOESM1_ESM.pdf]

Title: **Regulation of filial imprinting and structural plasticity by mTORC1 in newborn chickens**

Authors: Gervasio Batista[1], Jennifer L Johnson[2], Elena Dominguez[1], Mauro Costa-Mattioli[2], Jose L Pena[1]

Affiliations:

1-Department of Neuroscience, Albert Einstein College of Medicine. Bronx, New York.

2- Department of Neuroscience, Baylor College of Medicine. Houston, Texas.

Corresponding author:

Correspondence to: Jose Pena (jose.pena@einstein.yu.edu) and Gervasio Batista (gervasio.batista@phd.einstein.yu.edu)

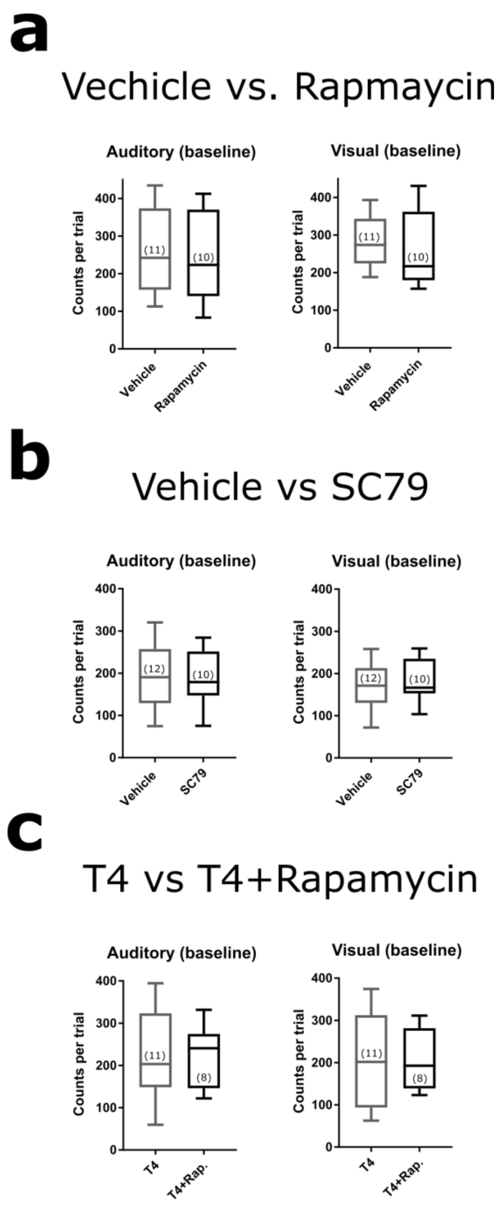

Suppplementary figure S1- Basal locomotion measures across experimental groups. No statistically significant differences in basal locomotion were found for the following comparisons: (a) P1, vehicle vs. rapamycin, (b) P4, vehicle vs. SC79 and (c) Thyroxine (T4) vs T4+Rapamycin. Box plots show mean and interquartile. Error bars show 10-90 percentiles. Number of animals used for each group is inside parenthesis

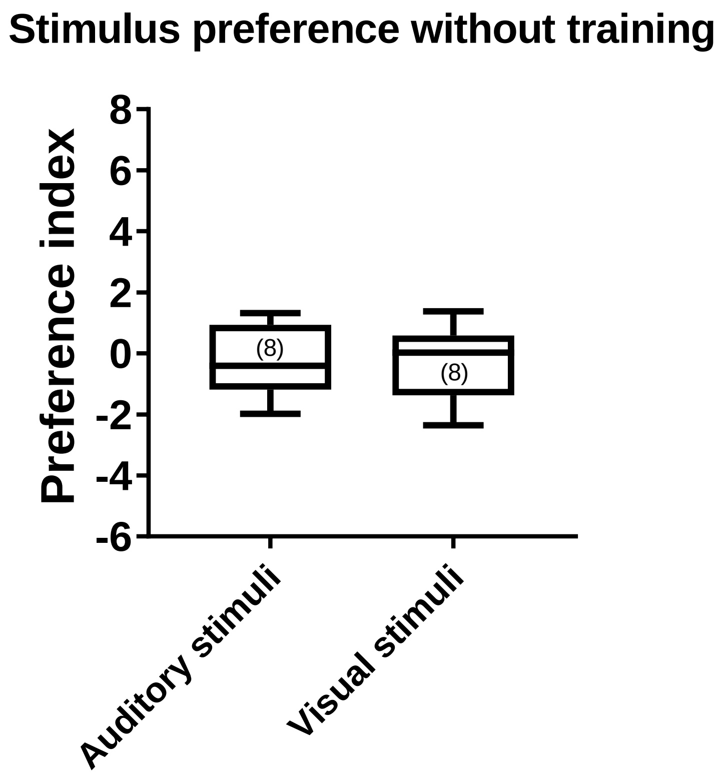

Supplementary figure S2- Preference index for auditory and visual stimuli of chicks without training. Chicks do not have an innate bias for any the stimuli used for this study ($N_{Auditory}$=8, N_{Visual}=8). One sample t-test, $p_{Auditory\ stimuli}$= 0.5543, $p_{Visual\ stimuli}$= 0.5263.
